# Anterior cingulate cortex activity as a candidate biomarker for treatment selection in social anxiety disorder

**DOI:** 10.1192/bjo.2018.15

**Published:** 2018-05-11

**Authors:** Andreas Frick, Jonas Engman, Kurt Wahlstedt, Malin Gingnell, Mats Fredrikson, Tomas Furmark

**Affiliations:** Department of Psychology, Uppsala University, Sweden and Department of Psychology, Stockholm University, Sweden; Department of Psychology, Uppsala University, Sweden; Department of Psychology, Uppsala University, Sweden and Department of Neuroscience, Uppsala University, Sweden; Department of Psychology, Uppsala University, Sweden and Department of Clinical Neuroscience, Karolinska Institutet, Sweden; Department of Psychology, Uppsala University, Sweden

**Keywords:** Functional magnetic resonance imaging, anxiety, prediction, selective serotonin reuptake inhibitors, cognitive–behavioural therapy, social phobia

## Abstract

We aimed to identify biomarkers to guide the decision to add selective serotonin reuptake inhibitors (SSRI) to psychological treatment for social anxiety disorder (SAD). Forty-eight patients with SAD underwent functional magnetic resonance imaging and collection of clinical and demographic variables before treatment with cognitive–behavioural therapy, combined on a double-blind basis with either escitalopram or placebo for 9 weeks. Pre-treatment neural reactivity to aversive faces in the dorsal anterior cingulate cortex (ACC), but not clinical/demographic variables, moderated clinical outcomes. Cross-validated individual-level predictions accurately identified 81% of responders/non-responders. Dorsal ACC reactivity is thus a potential biomarker for SAD treatment selection.

**Declaration of interest:**

None.

Cognitive–behavioural therapy (CBT) is often combined with selective serotonin reuptake inhibitors (SSRIs) to treat depression^1^ and anxiety,^2^ but the additional efficacy of this combination is debated.^3^^,^^4^ Indeed, for some patients, CBT may be sufficient, and adding further treatment will not increase the effect. Adherence to pharmacotherapy may also be reduced by patient preference and SSRI side-effects. Refined models of treatment selection for individual patients are therefore needed. Indeed, basing treatment choice on personal characteristics of the individual patient is one of the goals of precision psychiatry.^5^ A recent study showed that pre-treatment brain metabolism could differentially predict outcomes of CBT and SSRI monotherapies for depression,^6^ indicating the potential of using neuroimaging biomarkers for such treatment selection. However, it is not known whether this extends to combination therapies (SSRI + CBT) and anxiety disorders. Hence, we sought to conceptually replicate these findings^6^ to identify biomarkers that could guide decisions on whether to add SSRI medication to CBT in patients with social anxiety disorder (SAD). Based on previous treatment response prediction studies,^7^^,^^8^ we hypothesised that activity in the amygdala and anterior cingulate cortex (ACC) would be predictive of treatment response.

## Method

This study relates baseline neural, demographic, and clinical data to treatment outcome reported in a previous double-blind randomised controlled trial.^3^ For a detailed description of participant recruitment, treatment, demographic/clinical measures, neuroimaging pre-processing and first-level analyses, refer to the original publication.^3^ Briefly, 48 patients with SAD (mean ± SD age 33.2 ± 8.8 years, 24 women) were treated for 9 weeks with internet-based CBT, combined either with the SSRI escitalopram (20 mg) or a pill placebo. The primary outcome measures were treatment response category as measured by the Clinical Global Impression Improvement scale (responders ≤ 2; non-responders ≥ 3) and symptom improvement assessed with the clinician-administered Liebowitz Social Anxiety Scale.^9^ The participants also underwent functional magnetic resonance imaging during a disorder-relevant emotional face-matching task with shape-matching control trials,^3^ and were assessed regarding demographic/clinical variables age, gender, symptom severity, duration and subtype of SAD, comorbidity, previous treatment, and depression level.

To examine how pre-treatment brain reactivity (faces minus shapes) and demographic and clinical variables moderated the effect of the treatment group (SSRI + CBT or placebo + CBT) on clinical response category and symptom improvement, we conducted separate regression analyses for each outcome measure and for each variable of interest using the *glm* function in R.^10^ Each voxel and demographic/clinical predictor variable was thus entered into a separate regression model, together with the treatment group and interactions between the variable and the treatment group. The interaction term was our focus here, as it is a measure of differential prediction of clinical outcome in the two treatment groups (i.e. moderation). The threshold for voxel-wise brain analyses was set at *P* < 0.005 with a cluster size >10 voxels, to balance type I and type II errors.^11^ For demographic and clinical variables, we used the standard *P* < 0.05 threshold for significance. It should be noted that, in order to be stringent, we required moderation of both outcome measures at these statistical thresholds.

The study was approved by the Regional Ethical Review Board, Uppsala, and the Medical Products Agency in Sweden. All participants were fully informed about the study aims and procedures and gave written informed consent prior to inclusion.

## Results

**Fig. 1:**
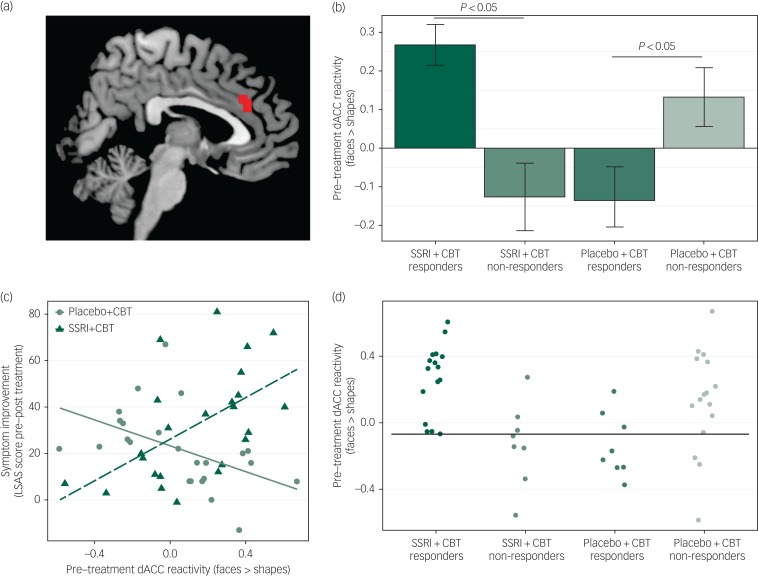
(a) Cluster in the dorsal anterior cingulate cortex (dACC) where pre-treatment neural reactivity to an emotional face-matching task moderated the effect of treatment group on clinical response category and continuous symptom improvement. The cluster is overlaid on a standard anatomical brain image. (b) Bar plot illustrating that responders to SSRI + CBT had increased pre-treatment dACC reactivity (faces > shapes) relative to non-responders, whereas responders to placebo + CBT had reduced dACC reactivity as compared to non-responders. Error bars denote standard error of the mean. (c) Differential correlations between pre-treatment reactivity in the dorsal anterior cingulate cortex (dACC) and symptom improvement [change (pre–post) on the Liebowitz Social Anxiety Scale (LSAS)] between groups. In the SSRI + CBT group, there was a positive correlation and in the placebo + CBT group a negative correlation. (d) Illustration of treatment response predictions at the individual patient level based on pre-treatment dACC reactivity (faces > shapes). The horizontal black line denotes the optimal threshold, maximizing classification accuracy. SSRI + CBT responders and placebo + CBT non-responders above the threshold and SSRI + CBT non-responders and placebo + CBT responders below the threshold were correctly classified, in total 81%.

Pre-treatment neural reactivity (faces > shapes) in the dorsal ACC (dACC; cluster size 648 mm^3^) differentially predicted both the clinical response and the symptom improvement outcome variables in the two treatment groups (Figure 1). Pre-treatment dACC reactivity was higher in responders (*n* = 16) than in non-responders (*n* = 8) (*t*(22) = 4.06, *P* = 0.0005) in the SSRI + CBT group, whereas the reverse was true in the placebo + CBT group, i.e. there was lower reactivity in responders (*n* = 8) than in non-responders (*n* = 16) (*t*(22) = 2.25, *P* = 0.035) (Figure 1b). Accordingly, higher pre-treatment dACC reactivity predicted symptom improvement in the SSRI + CBT group (*r*(22) = 0.59, *P* = 0.002), but worse outcome in the placebo + CBT group (*r*(22) = −0.52, *P* = 0.009) (Figure 1c). No other neural or demographic/clinical moderating variables were identified.

The predictive accuracy of pre-treatment dACC reactivity for individual patients was examined by applying a reactivity threshold (β = 0) based on mean β values (faces minus shapes) from the dACC cluster, i.e. individuals with high dACC reactivity (β > 0) were predicted to respond to SSRI + CBT but not to placebo + CBT, and *vice versa* for individuals with low dACC reactivity. Accuracy was calculated as the ratio of participants correctly identified as responders or non-responders. This arbitrary threshold resulted in 75% accurate predictions (high reactivity: 86%; low reactivity: 60%). We also calculated the optimal reactivity threshold (β = −0.068) using leave-one-subject-out cross-validation, to maximise predictive accuracy in this sample while at the same time taking generalisation to other samples into account, which resulted in 81% accurate predictions (high reactivity: 83%; low reactivity: 77%) (Figure 1d).

## Discussion

Pre-treatment neural activity to emotional faces in the dACC predicted clinical outcome to CBT when combined with either an SSRI or placebo. Specifically, highly reactive individuals were more likely to respond to SSRI-augmented CBT but not to placebo-paired CBT; conversely, lower reactivity was associated with response to combined placebo + CBT and non-response to SSRI + CBT. These results are in line with a recent report on unmedicated SAD patients showing lower pre-treatment dACC reactivity in CBT responders than in non-responders,^8^ and also with previous studies indicating that neural reactivity in the ACC is predictive of treatment response in depression and anxiety disorders.^7^^,^^12^ The dACC is hyper-reactive in SAD patients compared with healthy controls^13^ and has a key role in many functions that are affected by SAD, including fear expression and emotion regulation.^14^ The interaction between dACC reactivity and treatment (SSRI + CBT or CBT) may thus suggest that the two treatments differentially tax such functions. Contrary to our hypothesis, pre-treatment amygdala reactivity did not predict treatment response. This may be somewhat surprising given previous reports of a change–change relationship between reduced amygdala reactivity with treatment and symptom improvement, which was also observed in the current sample.^3^ Superior treatment prediction from neural as opposed to demographic/clinical variables is, however, consistent with previous studies on monotherapy.^7^^,^^8^ Among the limitations, it should be noted that the sample size was small, and the results should be regarded as tentative until replicated. In conclusion, pre-treatment dACC reactivity, but not demographic/clinical characteristics, predicted who would benefit from adding SSRI to CBT. In line with the goals of precision psychiatry, these results support dACC reactivity as a putative biomarker for treatment selection at the individual level, and suggest that brain imaging could improve clinical decision-making.
